# Long-term outcome of partial P450 side-chain cleavage enzyme deficiency in three brothers: the importance of early diagnosis

**DOI:** 10.1530/EJE-19-0696

**Published:** 2020-01-09

**Authors:** Wafa Kallali, Ewan Gray, Muhammad Zain Mehdi, Robert Lindsay, Louise A Metherell, Federica Buonocore, Jenifer P Suntharalingham, John C Achermann, Malcolm Donaldson

**Affiliations:** 1 Children’s Hospital El Bechir Hamza of Tunis, Tunis, Tunisia; 2 David Elder Medical Practice, Glasgow, UK; 3 Pathology Department, Glan Clwyd Hospital, Rhyl, UK; 4 Institute of Cardiovascular and Medical Sciences, British Heart Foundation Glasgow Cardiovascular Research Centre, University of Glasgow, Glasgow, UK; 5 Centre for Endocrinology, William Harvey Research Institute, Queen Mary University of London, London, UK; 6 Genetics & Genomic Medicine, UCL Great Ormond Street Institute of Child Health, University College London, London, UK; 7 Child Health Section of University of Glasgow School of Medicine, Queen Elizabeth University Hospital, Glasgow, UK

## Abstract

**Objective:**

*CYP11A1* mutations cause P450 side-chain cleavage (scc) deficiency, a rare form of congenital adrenal hyperplasia with a wide clinical spectrum. We detail the phenotype and evolution in a male sibship identified by HaloPlex targeted capture array.

**Family study:**

The youngest of three brothers from a non-consanguineous Scottish family presented with hyperpigmentation at 3.7 years. Investigation showed grossly impaired glucocorticoid function with ACTH elevation, moderately impaired mineralocorticoid function, and normal external genitalia. The older brothers were found to be pigmented also, with glucocorticoid impairment but normal electrolytes. Linkage studies in 2002 showed that all three brothers had inherited the same critical regions of the maternal X chromosome suggesting an X-linked disorder, but analysis of *NR0B1* (DAX-1, adrenal hypoplasia) and *ABCD1* (adrenoleukodystrophy) were negative.

In 2016, next-generation sequencing revealed compound heterozygosity for the rs6161 variant in *CYP11A1* (c.940G>A, p.Glu314Lys), together with a severely disruptive frameshift mutation (c.790_802del, K264Lfs*5). The brothers were stable on hydrocortisone and fludrocortisone replacement, testicular volumes (15–20 mL), and serum testosterone levels (24.7, 33.3, and 27.2 nmol/L) were normal, but FSH (41.2 µ/L) was elevated in the proband. The latter had undergone left orchidectomy for suspected malignancy at the age of 25 years and was attending a fertility clinic for oligospermia. Initial histology was reported as showing nodular Leydig cell hyperplasia. However, histological review using CD56 staining confirmed testicular adrenal rest cell tumour (TART).

**Conclusion:**

This kinship with partial P450scc deficiency demonstrates the importance of precise diagnosis in primary adrenal insufficiency to ensure appropriate counselling and management, particularly of TART.

## Established facts

- Primary adrenal insufficiency (PAI) is a life-threatening disorder of the adrenal cortex, characterised by impaired production of glucocorticoids with or without deficiency in mineralocorticoids and adrenal androgens.
- Classic *CYP11A1* mutation causes under-androgenisation in affected 46,XY subjects and severe salt-wasting in both 46,XY and 46,XX individuals due to disruption of the P450 side-chain cleavage (P450scc) enzyme.
- Partial P450scc deficiency presents with late-onset primary adrenal insufficiency (PAI) without genital anomalies.
- Partial P450scc deficiency is emerging as a surprisingly common cause of previously undiagnosed PAI.

## Novel insights

- P450scc deficiency was discovered only in adulthood in three brothers who had presented during childhood with glucocorticoid deficiency, modest mineralocorticoid impairment, and intact Leydig cell function and who had been labeled as having PAI of unknown cause.
- One sibling had undergone radical left orchidectomy at 25 years for suspected malignancy. Histology had initially been reported as ‘Leydig cell hyperplasia’, but the diagnosis was revised to testicular adrenal rest cell tumour (TART) in the light of the recent diagnosis of P450scc deficiency, with positive CD56 staining in adrenal rest cell nodules and clumps, but not in the Leydig cells.
- This sibship highlights the importance of considering rare forms of congenital adrenal hyperplasia in the differential diagnosis of adrenal insufficiency.

## Introduction

The term primary adrenal insufficiency (PAI) describes a group of potentially life-threatening disorders, either congenital or acquired, in which an intrinsic defect results in impaired cortisol synthesis. The term ‘Addison’s disease’ is often used as a synonym for PAI, although Addison’s original paper described adrenal failure in six patients with tuberculosis, hence, some workers reserve the term for acquired PAI (1).

Congenital PAI is usually due to monogenic causes (2, 3). Principal causes include congenital adrenal hyperplasia (CAH), X-linked congenital adrenal hypoplasia due to disruptions in NR0B1 (DAX-1), familial glucocorticoid deficiency (FGD) and related ACTH-resistance conditions, X-linked adrenoleukodystrophy (ALD), and Allgrove syndrome (Triple A syndrome).

Acquired PAI is usually due to autoimmune adrenalitis, either alone or in combination with other autoimmune disorders (2, 4, 5, 6, 7). Tuberculous destruction of the adrenal glands remains an important cause of PAI worldwide (8).

Recent advances in genetic investigations have contributed to the determination of the underlying etiology in more than 80% of children with PAI (9, 10, 11).

Recently, there has been growing interest in PAI due to one rare form of CAH – cholesterol side-chain cleavage enzyme or P450scc deficiency. The P450scc enzyme plays a key role in the initial steps of steroidogenesis by catalyzing the conversion of cholesterol to pregnenolone in steroidogenic tissues such as the adrenal gland and gonads and is encoded by the *CYP11A1* gene, localized on chromosome 15q23-q24 (12).

P450scc deficiency (OMIM 613743) causes impaired production of gonadal and adrenal steroids and may result in complete or partial adrenal insufficiency with a wide range of clinical manifestations. To date, fewer than 40 patients with P450scc deficiency have been reported. Classic deficiency presents with severe early-onset adrenal insufficiency in the neonatal period and female external genitalia in 46,XY individuals (11, 13, 14, 15, 16, 17, 18, 19). Partial defects result in late-onset adrenal insufficiency or glucocorticoid insufficiency alone, associated with either normal genitalia or variable degrees of underandrogenisation (11, 15, 19, 20, 21, 22, 23, 24, 25).

Recently, Maharaj and colleagues reported that the missense mutation rs6161, c.940G>A, p.Glu314Lys, previously predicted to be a benign variant, results in PAI in the context of compound heterozygosity when combined with variations harboring disruptive changes (26). In all, 28 cases of P450scc deficiency related to the presence of the rs6161, c.940G>A, p.Glu314Lys variant have been described, 19 by Maharaj et al (26) and 9 from other centres (19, 24, 25).

In this report, we detail the diagnosis and outcome in Family 8 of the Maharaj paper, comprising three brothers, one of whom developed testicular adrenal rest tumor (TART). We also explore the question of gonadal impairment in P450scc deficiency, particularly relating to fertility.

## Patients and methods

**Figure 1:**
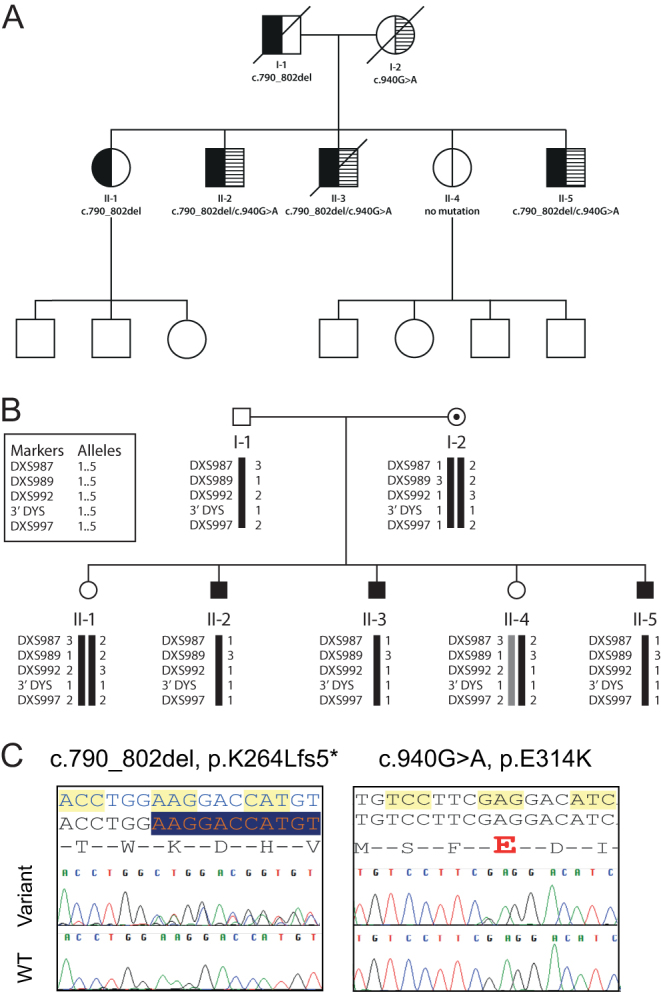
(A) Family tree of three generations in a non-consanguineous Scottish kinship with CYP11A1 deficiency affecting three brothers. The father carries a disruptive variant (c.790_802del, K264Lfs*5), while the mother is a carrier for a relatively common variant (c.940G>A, p.Glu314Lys) which was previously predicted as benign but which has been found to affect splicing. All brothers are compound heterozygotes, one sister is a carrier for c.790_802del mutation and the other is unaffected. None of the third generation has been tested. (B) X-linkage studies in a kinship with primary adrenal insufficiency indicating that the three affected brothers had inherited the same maternal X chromosome, suggesting an X-linked disorder. Subsequently, the brothers were found to be compound heterozygotes for CYP11A1 deficiency, inherited in autosomal recessive fashion. (C) Chromatogram showing paternally inherited c.790_802del, K264Lfs*5 and maternally inherited c.940G>A, p.Glu314Lys changes in *CYP11A1*, resulting in P450 side-chain cleavage enzyme deficiency.

Figure 1A shows three generations of a Scottish family featuring the father (I-1), and mother (I-2), their five children – two daughters (II-1 and II-4), three sons (II-2, II-3, II-5), and the offspring of the II-1 and II-4.

DNA was collected from both parents and all five children in 2002, following written informed consent in order to carry out X-linkage studies and DAX-1 analysis. The remaining DNA was subsequently stored.

In 2016, a consent was obtained once again from the five children, the parents being now deceased, for further molecular genetic analysis on the already stored DNA. Next-generation sequencing studies were performed as part of an initiative to assess the prevalence of CYP11A1 deficiency in PAI in 2016. In this study, HaloPlex targeted Gene Panel analysis was performed as described (26).

Following this, consent was obtained from II-5 for information on testicular surgery and fertility treatment in separate hospitals outside Scotland to be gathered. Finally, DNA from I-1 and II-4 were reanalyzed in 2019 after further DNA had been sent from Glasgow, since the previous sent samples had been insufficient. Testing of the third generation was not undertaken.

## Family study

### Childhood presentation

The proband (II-5) was born at 38 weeks of gestation by caesarean section for placental abruption weighing 2.84 kg and presented initially with poor feeding and weight faltering at 5 months. He was then admitted collapsed, mottled and cyanosed at the age of 21 months with a 1-day history of diarrhoea and fever. On examination, weight was 7.8 kg, s.d. was 4.31 below the mean, length was 75 cm (−2.95 s.d.). Investigations showed normal sodium (138 mmol/L) and potassium (3.9 mmol/L), with a compensated metabolic acidosis (pH 7.38, bicarbonate 13 mmol/L, base excess −15) and hypoglycaemia (blood glucose 1.6 mmol/L). A clinical diagnosis of septicaemia was made. He was given 10% dextrose with 1/5th normal saline at 50 mL/h initially (10.7 mg/kg/min of glucose) after which no further hypoglycaemia occurred. He was ventilated for 12 days, given Cefotaxime and Metronidazole, and fluid support with Dopamine but did not receive steroids at any point. He had an acute respiratory distress syndrome with left pneumothorax but went on to make a full recovery and was discharged at 6 weeks. Blood cultures and viral studies were negative. He was readmitted shortly after this with cough and fever (38.6°C) accompanied by a 1-min episode of stiffness, staring and unresponsiveness (Table 1).

**Table 1:** Details of childhood presentation, treatment and pubertal status in three brothers with partial P450scc deficiency due to a *CYP11A1* mutation (rs6161 variant (c.940G>A, p.Glu314Lys) affecting splicing and another disruptive variant causing frameshift and premature stop codon (c.790_802del, K264Lfs*5)).

	II-2	II-3	II-5
Birth year	1979	1980	1985
Birthweight (kg)	2.6	2.52	2.84
Gestation (weeks)	40	40	38
Age at diagnosis (years)	8.9	8	3.7
Height (cm) (SDS) at diagnosis	125 (−1.3)	117 (−2.0)	94 (−1.5)
Weight (kg) (SDS) at presentation	22 (−1.78)	18.5 (−2.67)	13.8 (−1.13)
Initial investigations			
Basal/peak cortisol (nmol/L)	278/289	339/389	174/178
Lowest plasma sodium (mmol/L)	137	138	128
Highest plasma potassium (mmol/L)	4.7	4.2	5.9
ACTH (mU/L) (*n* < 20)	N/A	N/A	1089
Renin (µIU/mL) NR (9–50)	N/A	N/A	1209
Maintenance treatment one year after diagnosis			
Hydrocortisone (mg/m^2^/day)	12	21.5	16.6
Fludrocortisone (µg/day)	100	100	100
Growth and pubertal status			
Age at G2	13 years	14 years	12 years
Age at G4	15.8	16.0	14.1
Testicular volume at G5	12–15 mL	12–15 mL	15 mL
Final height (cm)(SDS)	171 (−0.8)	168 (−1.2)	171 (−0.8)

At the age of 3.7 years, the child was admitted with fever (39.5°C) and tonsillitis associated with a 15-min tonic-clonic convulsion. On this occasion he was noted to be pigmented and investigations showed a flat cortisol response to a synthetic ACTH (Synacthen) test under fasting conditions, with elevated renin at 1209 μIU/mL (normal <50) and ACTH at 1089 mU/L (normal <20), and hyponatraemia (128 mmol/L) (Table 1) indicating primary adrenal insufficiency. Blood glucose was 2.8 mmol/L. He was started on treatment with Hydrocortisone and Fludrocortisone (Table 1).

Two further acute admissions while on steroid replacement occurred at the age of 4.5 and 5.7 years, both featuring fever and convulsions, with normal electrolytes and glucose (4.3 mmol/L) on the first occasion.

At the time the proband was diagnosed with PAI, both brothers were also noted to be pigmented. Synacthen tests were performed, therefore, which confirmed PAI (Table 1). Electrolytes were not recorded at this time. Subsequent electrolytes did not show hyponatraemia or hyperkalaemia but subsequent ACTH and renin values while on treatment showed elevation: renin 73 μU/mL and ACTH 1532 mU/L for II-2; and renin 120 μIU/mL and ACTH 66 mU/L (suspected degradation of sample) for II-3.

None of the brothers showed evidence of salt-craving although the family diet was noted to be high in crisps.

#### Family history

Parents were non-consanguineous. I-1 had undergone valve replacement for aortic valve disease. I-2 experienced convulsions in childhood until the age of 7, suffered from alcohol dependency, and was judged to have a degree of learning impairment.

II-1 and II-4 were both born at 40 weeks, weighing 3.1 and 2.6 kg. II-1 had a history of febrile convulsion at the age of 2 years and has developed aortic valve disease requiring replacement in adult life.

II-2 had several convulsions with fever in early childhood and was treated with phenobarbitone. He was hypotonic in infancy and showed developmental delay, not walking until 3 years or speaking clearly until 5 years of age.

II-3 had been hospitalized after birth for respiratory distress and hyperbilirubinemia. He was hypotonic with delayed motor milestones. At the age of 3 years he had a 30-min convulsion (temperature 38.2°C) and a further 2-min fit with fever at the age of 4 years.

Both II-2 and II-3 had tested negative for Fragile X syndrome and Duchenne muscular dystrophy. Both brothers were attending the same special educational needs school at the time II-5 was diagnosed.

#### Child and adolescent follow-up

None of the brothers were hospitalized for acute adrenal insufficiency. A target glucocorticoid dose of hydrocortisone 12 mg/m^2^/day was given and then adjusted according to clinical features including general health and well-being, growth rate, and BMI. As shown in Table 1, II-2 was receiving the expected glucocorticoid dose after 1 year, whereas the hydrocortisone doses in II-5 and II-3 had been increased on clinical grounds, for example, thin build – to 16.6 and 21.5 mg/m^2^/day respectively. II-3 remained deeply pigmented throughout childhood and adolescence despite receiving a supraphysiological replacement dose and parental assertion that he was the better tablet-taker of the three brothers.

Linear growth was satisfactory in all three brothers and all completed puberty with normal testicular volumes, although II-2 and II-3 entered puberty 2 years later than average. BMI values were 15.96, 15.3, and 15.44 kg/m^2^ for II-2, II-3, and II-5 at 12 years of age, indicating that a low BMI was not responsible for the pubertal delay in II2 and II-3. In fact, the elder brothers had higher BMI than II-5 at 14 years of age – 18.82, 17.45, and 14.38 kg/m^2^, respectively. Final heights were in the lower half of the parental target range – mid-parental height 174.5 cm (−0.3 SDS) (Table 1).

#### Initial genetic studies (2002)

Because of the likelihood of an X-linked disorder affecting the three brothers, markers were used to determine which X-chromosome had been inherited by each member of the second generation. Four overlapping primer sets covering the two exons of the *NR0B1* (DAX-1) gene were then used to sequence the middle brother’s DAX-1 gene: DX1, DX2, DX3, and DX4. Figure 1B shows that all three brothers and the sibling II-4 have inherited the same maternal sex chromosome. In the light of the clinical presentation, family pattern and X-linkage findings, X-linked PAI was considered likely but genetic analysis for *NR0B1* (DAX-1) was negative, while very long chain fatty acid and studies were normal, excluding X-linked adrenoleukodystrophy. Genetic studies were also carried out in Germany (Professor Angela Hübner, Children’s Hospital, Medical Faculty, Technical University Dresden, Germany) to exclude Triple A syndrome and showed normal results.

### Adult outcome

In 2016, Haloplex genetic analysis revealed compound heterozygosity of the *CYP11A1* gene with a disruptive variant causing frameshift and premature stop codon (c.790_802del, K264Lfs*5) and another relatively common variant rs6161 (c.940G>A, p.Glu314Lys) which was previously predicted ‘benign’ but was found to affect splicing (Fig. 1C).

The elder sister II-1 had developed aortic valve disease requiring replacement as had a paternal half-brother. It was noted that brothers II-2 and II-3 were suffering from alcohol dependency and that II-3 was receiving treatment for epilepsy with slow-release Sodium valproate.

#### Clinical and laboratory assessment of the three brothers

At clinical review aged 32, 36, and 37 years, the brothers were noted to be stable on hydrocortisone and fludrocortisone replacement. Glucocorticoid doses were high – 17.3, 18.6, and 24.6 mg/m^2^/d – but with no clinical features to indicate overreplacement, slight pigmentation, and normal BMI (Table 2).

**Table 2:** Data of three adult brothers with *CYP11A1* mutation (rs6161 variant (c.940G>A, p.Glu314Lys) affecting splicing and another disruptive variant causing frameshift and premature stop codon (c.790_802del, K264Lfs*5)).

	II-2	II-3	II-5
Age at evaluation (years)	37.3	36.4	31.6
Weight (kg)	65.6	58.3	58.5
BMI (kg/m^2^)	22.4	20.6	20
Blood pressure (mm Hg)	112/66	152/94	106/58
Tanner stage Testicular volumes R/L	G5P5 20/20	G5P5 15/15	G5P5 20/–
Investigations			
Basal FSH IU/l (1–12)	13.8	9.3	41.2
Basal LH IU/l (0.6–12.1)	5.8	5.4	33.9
Serum testosterone (nmol/L) (8.3–33)	24.7	33.3	6
ACTH (mU/l) (<20)	1532	43	1267
Renin (ng/mL/h) (<2.6)	12.6	13.2	27.2
Maintenance treatment			
Hydrocortisone, mg (mg/m^2^/day)	30 (17.3)	30 (18.6)	40 (24.6)
Fludrocortisone (µg/day)	100	100	200
Adherence	Good (6/7 days)	Moderate (forgets most mornings)	Poor until 2 years ago (see text)

Hormone reference ranges for Queen Elizabeth Glasgow Hospitals is given in brackets. Please see Table 1 for final height data.

There was scanty body hair but normal pubic hair, normal testicular volumes (15–20 mL), and normal serum testosterone. However, FSH values were borderline high in II-2 and frankly elevated in II-5. Samples for Inhibin B measurement were received by the laboratory but not analysed, owing to a system failure.

It also transpired that the proband (II-5) had presented to a hospital outside Scotland during the previous year with a 4-year history of left testicular swelling and discomfort. Review of medical records indicated that ultrasound had shown bilateral varicoceles, a left epididymal cyst, and a suspicious 6 × 7 mm mass at the upper of the left testis. Testicular tumour marker lactic dehydrogenase was 600 µ/L (reference range 256–500). At a multidisciplinary meeting, the diagnosis of ‘Addison’s disease’ was noted and radical left inguinal orchidectomy was scheduled. The initial histology report indicated ‘nodular Leydig cell hyperplasia.’

After review in 2017, II-3 died during an epileptic convulsion which followed a 48-h period of binge drinking. Post-mortem examination was not requested by the Procurator Fiscal.

**Figure 2:**
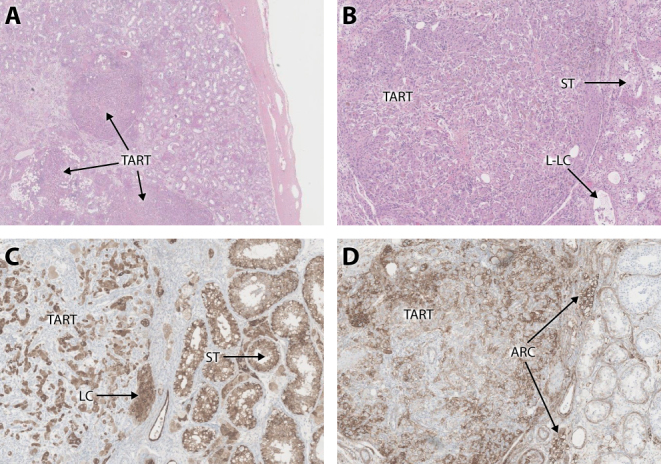
Testicular histology following radical orchidectomy for suspected malignancy in a subject with testicular rest cell tumour (TART) secondary to poorly controlled congenital adrenal hyperplasia due to CYP11A1 deficiency. Staining with haematoxylin and eosin (A and B) shows the nodules of TART. Inhibin staining (C) is positive for TART but also Leydig cells (LC), seminiferous tubules (ST), and lipid-laden cells (L-L C), believed to be adrenal rest cells. Staining with CD56 (D) shows uptake by some cells in the TART nodule and adrenal rest clumps (ARC) but not the Leydig cells or seminiferous tubules.

In 2019, the hospital responsible for II-5’s orchidectomy were contacted and asked to review the testicular histology. Figure 2A and B show nodules of TART on H&E low power. Staining with Inhibin (Fig. 2C) is non-specific. However, staining with CD56 (neural cell adhesion molecule 1) (Fig. 2D) shows patchy uptake in the nodules and in clumps of lipid-laden interstitial cells, but no uptake by seminiferous tubules or Leydig cells, consistent with nodules and clumps of adrenal rest cells. A Johnsen count performed on the spermatozoa on histology showed a reduced score of 7.5 – normal adult score 8.9 (27) – indicating decreased spermatogenesis.

II-5 retrospectively rated his adherence with hydrocortisone and fludrocortisone since childhood as very poor from 12 to 17 years of age, patchy from 17 to 29 years, and good during the previous 3 years.

Further enquiry revealed that II-5 and his partner had been trying for a baby for 4 years and had attended a fertility center for assessment. Semen analysis had shown reduced sperm concentration – 2.0 million per mL (normal 15), reduced sperm count at 8 million (normal 39 million per ejaculate), reduced motility with 77% immobile and 18% progressive (normal ≥40% motile and ≥32% progressive), and reduced normal forms. The couple had been enrolled into an *in vitro* fertilisation programme.

## Discussion

This sibship demonstrates a relatively mild phenotype of CYP11A1 deficiency with late presentation, dominant glucocorticoid deficiency, mild mineralocorticoid deficiency, no disorder of sex development, and intact Leydig cell function.

It is hard to assess the clinical contribution of CYP11A1 deficiency to the sibship prior to diagnosis, given that features such as convulsions and learning difficulties had affected other family members. Although it is possible that the severity and duration of the proband’s acute illness requiring ventilation might have been worsened by the adrenal insufficiency, the fact that he recovered fully without steroid support indicates that this was not the primary cause.

The partial nature of P450scc deficiency in this family is consistent with most of the published cases with similar genetic findings (19, 24, 25, 26).

In the context of the three affected brothers, two of whom had learning difficulties, two unaffected sisters, a mother with probable learning difficulties, and the finding of inheritance of the same X chromosome in the three siblings, it is understandable that an X-linked disorder akin to DAX-1-associated adrenal hypoplasia was thought likely. In fact, the cause was an autosomal recessive disorder, and it was purely by chance that only the males were affected. The probability of all three boys and neither of the two girls being affected with P450scc deficiency is calculated at just under 1% – unlikely but not extremely unlikely – indicating that X-linked inheritance should not be assumed when only males are affected.

Glucocorticoid deficiency was the dominant feature of PAI in this sibship. All three brothers were significantly pigmented at presentation, with no significant rise in baseline cortisol levels after synthetic ACTH stimulation, indicating markedly impaired glucocorticoid synthesis and elevated ACTH levels, even in the absence of actual ACTH measurement at baseline in II-2 and II-3. The modest ACTH elevation seen thereafter in the older brothers, despite significant pigmentation, could reflect the problems with sample degradation which were encountered in our hospital in the late 1980s and early 1990s. Furthermore, ACTH concentrations may also vary considerably in the same individual, depending on when the hydrocortisone was taken and when the blood collection took place.

High hydrocortisone doses were given to the middle brother (II-3), who was always deeply pigmented and thin as if undertreated, and to the proband (II-5) who went on to develop TART. The doses administered (Table 1) considerably exceed the 10–12 mg/m^2^/day recommended in PAI (28). It should be noted that the degree of pigmentation is of limited usefulness as an index of cortisol replacement therapy and that over-reliance on this marker risks overtreatment, although this was not the case in our patients.

While poor compliance might seem an obvious explanation for the high hydrocortisone requirement, and was acknowledged by II-5 to have been very poor in adolescence and early adult life, the parents claimed good compliance during the childhood years; a view confirmed by the four siblings at interview in 2019 after II-3’s death. There is, therefore, no satisfactory explanation for this observation. It should be noted that persistently elevated ACTH can be seen in familial glucocorticoid deficiency (e.g. due to MC2R or ACTH receptor mutations) and attempts to suppress this with high replacement doses can potentially cause signs of glucocorticoid excess. However, this was not the case in II-3, who had a persistently low BMI.

By contrast, mineralocorticoid deficiency was mild in the sibship. Hyponatraemia was only recorded once – in the proband when he presented with a septicaemia-like illness. On this occasion, the sodium value of 128 mmol/L may not have been purely related to salt loss, and glucocorticoid deficiency *per se* may result in hyponatraemia by reducing water excretion (29). However, renin levels were elevated even on treatment in the patients, and crisps formed an important part of the diet. It could be argued that a smaller dose of mineralocorticoid could have been given, but it is our policy to give a full replacement dose of fludrocortisone, even with subclinical aldosterone deficiency to ensure adequate replacement, being prepared to reduce this in the event of hypertension and/or hypokalaemia, neither of which occurred.

Consistent with previous reports, pubertal development (although delayed in the two older brothers) was normal in the sibship, with normal testicular volumes and Leydig cell function (19, 24, 25, 26).

However, long-term follow-up has demonstrated abnormal germ cell function in the proband with FSH elevation, oligozoospermia, and reduced sperm motility. FSH elevation is probably related to disturbed Sertoli cell function secondary to development of TART (30, 31, 32), on a background of many years during which adherence to steroid therapy was poor.

TART is well described as a feature of poorly controlled 21-hydroxylase deficiency, the most common form of CAH (32, 33). Of note, angiotensin II receptors are found in TART tissue, so that mineralocorticoid deficiency, in addition to glucocorticoid deficiency, may contribute to its development (33).

However, factors other than poor hormonal control have been implicated in the development of TART; these include testicular ‘misplacement’ of adrenal cells during development, with prenatal exposure to increased ACTH levels, and the presence of pluripotent cells which can differentiate into adrenocortical cells after ACTH stimulation (34, 35).

TART has also been described in conditions other than 21-hydroxylase deficiency, with reports of this complication in rarer forms of CAH such as 11-hydroxylase deficiency and 3β-hydroxysteroid dehydrogenase type 2 deficiency but not in autoimmune PAI (32, 33).

There is only one previous report of TART in two peripubertal male patients with CYP11A1 deficiency (25). The diagnosis in these two reported patients was made by ultrasound, which is the preferred method of detection (33), while there are both ultrasound and histological data in the case we describe.

Our case demonstrates the histological features of adrenal rest cells in the removed testis and shows that the CD56 stain was the most discriminating in demonstrating that the cells in the nodules and the clumps of interstitial cells were adrenal in origin. By contrast, Inhibin staining was less specific for adrenal tissue while Melan staining did not identify the clumps of adrenal rest cells (data not shown). The CD56 findings are similar to that described by Ali* et al.* in which a 15-year-old boy with 21-hydroxylase deficiency underwent bilateral orchidectomy, the adrenal rest tissue showing strong staining for both CD56 and synaptophysin (36). Of note, CD56 staining demonstrated lipid-laden cells in our case. While macrophages may stain similarly, the site of these cells in the context of CYP11A1 deficiency strongly suggests that these are adrenal in origin. The increased lipid content is consistent with the proximal adrenal enzyme block and analogous to STAR protein deficiency. Consistent with this, a previous study in *Cyp11a1-null* mice demonstrated lipid accumulation in fetal adrenals, leading to the degeneration of adrenocortical cells soon after these cells had differentiated (37).

Reduced fertility is a recognised consequence of inadequately controlled 21-hydroxylase deficiency with TART formation but has not previously been documented in P450scc deficiency. It is likely that the fertility problems in our patient are related to TART with the impairment in sperm production, as indicated by the reduced Johnsen count, consistent with a toxic paracrine effect of the steroid hormones or metabolites produced by the adrenal rests as in 21-hydroxylase deficiency (32, 33, 38). A reduction in sperm count could also result from obstruction caused by the nodules and adrenal rest clumps compressing the rete testis and seminiferous tubules (32, 33, 38). Another possible explanation is that lipid accumulation in steroidogenic cells could also be an additional factor of reduced spermatogenesis, similar to previous reports of patients with STAR protein deficiency (39).

The missed diagnosis of TART as the cause for testicular swelling in the younger brother relates to the lack of a specific diagnosis for his PAI at the time of presentation with testicular discomfort and left testicular swelling. Had the specific diagnosis been reached earlier, surgery would not have been the treatment of choice. Intensifying glucocorticoid treatment may improve testicular function in the early stages of TART by suppressing ACTH secretion leading to reduction of the tumour size (32, 33). Regular monitoring of these patients with ultrasound is also recommended to make an early diagnosis and offer patients with TART semen cryopreservation. Surgery is only indicated to relieve pain and discomfort in patients with TART and does not restore fertility (32, 33).

This case of P450scc deficiency and previous reports on fertility in patients with CAH due to 21 hydroxylase deficiency and StAR protein deficiency (31, 40) reinforces the importance of early diagnosis. Timely diagnosis ensures that gonadal function will be monitored in the long term, with a view to optimizing fertility and offering treatment, including sperm banking in progressive cases. In established cases, serial LH, FSH, testosterone, Inhibin B, and semen analysis should be recommended regularly in addition to clinical examination and serial testicular ultrasounds for adrenal rests (31, 32, 40).

Finally, PAI should be regarded as a descriptive diagnosis requiring a specific explanation. We caution against using the term ‘Addison’s disease’ in congenital PAI, since this implies a valid diagnosis. Had the problem in our proband been listed as ‘congenital primary adrenal insufficiency of unknown cause’, a link between the adrenal and his testicular problem might have been considered and a change in management might have been brought about.

## Declaration of interest

The authors declare that there is no conflict of interest that could be perceived as prejudicing the impartiality of this case report.

## Funding

J C A is a Wellcome Trust Senior Research Fellow in Clinical Science (grants 098513/Z/12/Z and 209328/Z/17/Z) with research support from Great Ormond Street Hospital Children’s Charity (grant V2518) and the National Institute for Health Research, Great Ormond Street Hospital Biomedical Research Centre (grant IS-BRC-1215-20012). L A M is supported by funding from Barts Charity (grant MGU0438) and MRC (Project Grant MR/K020455/1). The views expressed are those of the authors and not necessarily those of the National Health Service, National Institute for Health Research, or Department of Health.
